# Mutation analysis of a Chinese family with oculocutaneous albinism

**DOI:** 10.18632/oncotarget.13109

**Published:** 2016-11-04

**Authors:** Xiong Wang, Yaowu Zhu, Na Shen, Jing Peng, Chunyu Wang, Haiyi Liu, Yanjun Lu

**Affiliations:** ^1^ Department of Laboratory Medicine, Tongji Hospital, Tongji Medical College, Huazhong University of Science and Technology, Wuhan 430030, China; ^2^ Department of Obstetrics and Gynecology, Tongji Hospital, Tongji Medical College, Huazhong University of Science and Technology, Wuhan 430030, China

**Keywords:** oculocutaneous albinism, tyrosinase, SLC45A2, mutation

## Abstract

Oculocutaneous albinism (OCA) is an autosomal recessive disorder characterized by either complete lack of or a reduction in melanin biosynthesis in the skin, hair, and eyes. OCA1, the most common and severe type, is caused by mutations in the tyrosinase (*TYR*) gene. In this study, we report a Chinese family with two members affected by OCA. Blood samples were collected from all family members. Genomic DNA was isolated from blood leukocytes, and all coding exons and adjacent intronic sequences of the *TYR* gene were examined for mutation analysis using polymerase chain reaction (PCR)-based sequencing. A pedigree chart was drawn, and clinical examinations and paraclinical tests were performed. Compound heterozygous mutations in *TYR* (c.832C>T and c.929_930insC, which resulted in p.Arg278* and p.Arg311Lysfs*7, respectively) were identified in the two patients with milky skin, white hair, photophobia, and reduced visual acuity, while other family members only carried one of two heterozygous mutations. In addition, a homozygous missense mutation c.814G>A (p.Glu272Lys) in the solute carrier family 45 member 2 (*SLC45A2*) gene was found in both patients and unaffected family members, suggesting that this may not be a causative mutation. The findings of this study expand the mutational spectrum of OCA. Compound heterozygous mutations (c.832C>T and c.929_930insC) in the *TYR* gene may be responsible for partial clinical manifestations of OCA, while the homozygous missense mutation c.814G>A (p.Glu272Lys) in the *SLC45A2* gene may not be associated with OCA.

## INTRODUCTION

Oculocutaneous albinism (OCA) is a congenital and autosomal recessive disorder with an estimated prevalence of 1/17,000 worldwide. OCA is characterized by complete or partial lack of pigment in the skin, hair, and eyes due to a deficiency in melanin biosynthesis and is accompanied by optic defects, such as nystagmus, strabismus, and photophobia [1, 2]. In patients with OCA, the pigmentation present in skin, hair, and eyes may range from none to normal levels depending on the specific subtype. Clinical diagnosis of OCA type is difficult due to the variable clinical phenotypes. Thus, molecular analyses will provide important insights into accurate diagnosis and genetic counseling [3].

OCA can be classified as nonsyndromic and syndromic OCA. Nonsyndromic OCA includes four types, i.e., OCA1 (MIM#203100), OCA2 (MIM#203200), OCA3 (MIM#203290), and OCA4 (MIM#606574), caused by mutations in the tyrosinase gene (*TYR*), *OCA2*, tyrosinase-related protein gene (*TYRP1*), and solute carrier family 45 member 2 gene (*SLC45A2*), respectively [4–6]. OCA1 and OCA2 are the two most frequent types of OCA, accounting for approximately 50% and 30% of cases, respectively [5, 7]. OCA1 could be further subgrouped into OCA1A and OCA1B. In OCA1A, *TYR* null mutations producing incomplete polypeptides result in a complete lack of melanin throughout the patient's life. In OCA1B, *TYR* mutations producing hypomorphic TYR enzymes cause retention of some enzyme activity, resulting in the development of some yellow hair pigments during the first few years of life and gradual accumulation of pigment in the skin, hair, and eyes over time [8–10]. Recently, in silico screening and molecular dynamics simulation (MDS) approaches have been widely used to identify the most probable mutations associated with OCA by computational prediction of mutant structures and consequences [11–15]. These methods may provide insights into the underlying molecular mechanisms involved in OCA.

In our current study, compound heterozygous mutations in *TYR* (c.832C>T and c.929_930insC, which resulted in p.Arg278* and p.Arg311Lysfs*7, respectively) were identified in the two patients who both showed complete lack of melanin formation in the skin, hair, and eyes, accompanied by nystagmus and photophobia. In addition, a homozygous missense mutation, c.814G>A (p.Glu272Lys), in the solute carrier family 45 member 2 (*SLC45A2*) gene was found in both patients and unaffected family members.

## RESULTS

### Clinical phenotype

**Figure 1:**
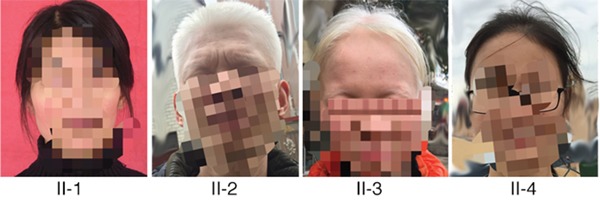
Clinical features of the families with OCA Patient 1 (II-2) and patient 2 (II-3) showed typical OCA1 symptoms in terms of skin, hair, and iris pigments (provided and mosaiced by II-4). II-1 and II-4 showed normal pigment formation in the skin, hair, and iris.

Clinical features of the two patients affected by OCA and two normal family members are shown in Figure 1. Both patients completely lacked pigmentation in the skin, hair, and eyes, even when they were adults, and presented with nystagmus and photophobia, showing typical symptoms of OCA1. In contrast, unaffected individuals in the OCA family exhibited normal pigment formation at the time of birth.

### Identification and analysis of mutations

**Figure 2:**
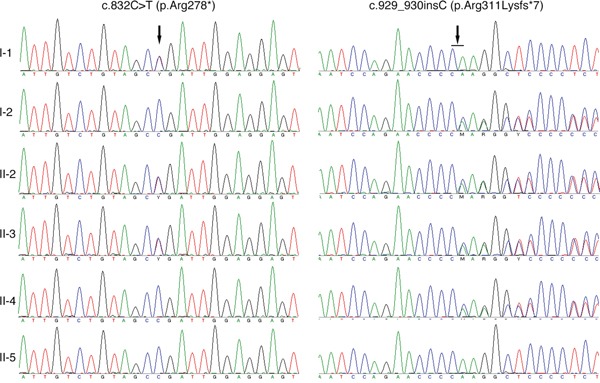
Sequencing results of the *TYR* gene I-1 is the father, and I-2 is the mother. II-1 and II-4 are the patients' older and younger sisters, respectively. II-5 is the spouse of II-4, and III-1 is their daughter.

**Figure 3:**
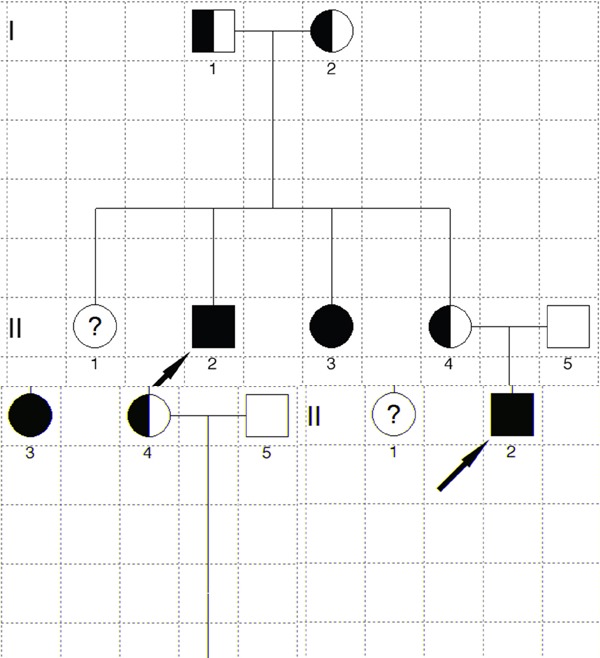
Pedigree of the OCA family The filled square marked with the arrow is the proband, and the filled circle is the patient. Half-filled squares or circles are carriers. Unfilled squares or circles denote unaffected family members. Question marks indicate that DNA analysis is unavailable. Squares represent males, and circles denote females.

The two affected patients both showed compound heterozygous mutations in *TYR* (c.832C>T and c.929_930insC). The *TYR* c.832C>T nonsense and c.929_930insC insertion mutations resulted in abnormal stop signals during translation (p.Arg278* and p.Arg311Lysfs*7, respectively). Their unaffected father harbored the heterozygous c.832C>T mutation, whereas their unaffected mother and younger sister both harbored heterozygous c.929_930insC mutations (Figure 2). A missense mutation in *SLC45A2* (c.814G>A) was found in both patients, which resulted in p.Glu272Lys (data not shown). However, homozygous *SLC45A2* c.814G>A was also found in unaffected family members, indicating that this mutation may not be the causative mutation. The mutations are summarized in Table 1, and the family pedigree was drawn (Figure 3). Because both patients showed typical OCA1 syndrome and compound heterozygous mutations in *TYR* were found, these data suggested that compound heterozygous mutations in *TYR* (c.832C>T and c.929_930insC) were associated with OCA1, whereas *SLC45A2* c.814G>A was not associated with OCA1.

**Table 1:** Mutation summary of the OCA family

Family member	c.832C>T (p.Arg278*)	c.929_930insC (p.Arg311Lysfs*7)
I-1	Heterozygous	-
I-2	-	Heterozygous
II-1	?	?
II-2 (Proband)	Heterozygous	Heterozygous
II-3	Heterozygous	Heterozygous
II-4	-	Heterozygous
II-5	-	-
III-1	?	?

## DISCUSSION

OCA1, with an estimated worldwide prevalence of 1/40,000, is caused by a mutation in *TYR*, which resides on chromosome 11q14.3 and encodes TYR protein. TYR has both tyrosine (a precursor to melanin synthesis) hydroxylase and dopa oxidase catalytic activities, which catalyze the critical first and second reactions, i.e., hydroxylation of tyrosine to l-3,4-dihydroxyphenylalanine (l-DOPA) and oxidation of l-DOPA to DOPA-quinone. A pseudogene known as the TYR-like gene (*TYRL*) is located on chromosome 11 and shares 98.55% sequence similarity within the 3′-region of *TYR* (exons 4 and 5) [16]. To avoid co-amplification of *TYRL*, locus-specific amplification was applied to amplify exons 4 and 5 of *TYR* as previously described [17]. Mutations in *TYR* can cause complete or partial OCA depending on residual activity. More than 200 different pathological mutations in *TYR* and 299 *TYR* mutations have been reported in the Albinism Database (http://www.ifpcs.org/albinism/index.html) and Human Gene Mutation Database (HGMD, http://www.hgmd.cf.ac.uk), respectively. In the clinical diagnosis of OCA, it is challenging to distinguish among different subtypes; however, as OCA is inherited autosomal recessively, molecular analysis is essential for accurate diagnosis of OCA.

In this study, we identified compound heterozygous mutations (c.832C>T and c.929_930insC) in *TYR* exon 2 in two Chinese patients by direct sequencing. Exons 1 and 2 of *TYR* contained mutational hotspots in the Chinese Han population [18]. The heterozygous mutation c.832C>T in *TYR* was first reported in Indo-Pakistani patients with OCA1 by Tripathi et al in 1993 [19], and homozygous c.832C>T was reported by Chaki et al in 2005 [20]. In the Chinese population, homozygous c.832C>T and heterozygous c.832C>T and c.929_930insC mutations in *TYR* were found in a screen of 127 unrelated Chinese patients with OCA in 2010 [18]. He et al and Wang et al had reported the mutations c.832C>T or c.929_930insC in *TYR* [21]. Both c.832C>T and c.929_930insC mutations in the *TYR* gene result in premature stop codons in the TYR protein and are thought to be pathogenic [21]. Moreover, in a study of five unrelated patients with OCA1, compound heterozygous mutations (c.832C>T and c.929_930insC) were observed [21], while another study showed that c.832C>T and c.929_930insC were present in different patients [24]. Our study reported an OCA family with both patients and their parents for the first time; furthermore, their unaffected sister was also genetically evaluated. Our work suggested that compound heterozygous mutations in *TYR* (c.832C>T and c.929_930insC) may have caused the OCA1 phenotype in the current pedigree.

We also screened mutations in *OCA2* and *SLC45A2*, and only *SLC45A2* c.814G>A (p.Glu272Lys) was found in patients with OCA. However, a homozygous *SLC45A2* c.814G>A mutation was found in unaffected family members, indicating that this mutation may be not associated with OCA1. This was consistent with a previous report and the patient's clinical phenotypes in the current OCA family as the *SLC45A2* mutation may contribute to OCA4 rather than OCA1.

In summary, we report an OCA family and the molecular basis of the disease pathogenesis identified by Sanger sequencing of all coding exons of *TYR*, *OCA2*, and *SLC45A2* genes. The findings of this study expand the mutational spectrum of OCA. Compound heterozygous mutations (c.832C>T and c.929_930insC) in the *TYR* gene may be responsible for partial clinical manifestations of OCA, while the homozygous missense mutation c.814G>A (p.Glu272Lys) in the *SLC45A2* gene may not be associated with OCA.

## PATIENTS AND METHODS

### Patient recruitment and ethical statement

One patient was a 34-year-old male, and the other patient was a 31-year-old female. Both patients showed typical features of OCA1. Written informed consent for genetic analysis and publication of personal photographs was obtained from each participant. This study was approved by the Medical Ethics Committee of Tongji Hospital, Tongji Medical College, Huazhong University of Science and Technology. All procedures were carried out in accordance with ethical guidelines for human subjects research. Family histories were determined, and pedigree charts were drawn to trace the inheritance model.

Detailed physical examinations and complete ophthalmic examinations were carried out, including slit-lamp examinations, best-corrected visual acuity testing, optical coherence tomography, and dilated fundus examinations.

### DNA extraction and mutational analysis

Genomic DNA was extracted using a QIAamp DNA blood mini kit (Qiagen, Hilden, Germany) from 200 μL peripheral blood. The primers were designed as described in the Master's Thesis of Peng Jie (Zhongnan University) or using Primer Premier 5.0; primers covered the sequences of all coding domains of *TYR*, *OCA2*, and *SLC45A2*, including exon/intron junctions, and primer sequences are shown in Table 2. The primers were synthesized by Invitrogen (Shanghai, China). Each 50-μL PCR mixture contained 100 ng genomic DNA, 2 μL of 10 μM forward and reverse primers (with a final concentration of 400 nM), and 25 μL of 2× Taq PCR MasterMix (Takara, Dalian, China). PCR was carried out in Veriti thermocycler (Applied Biosystems, Foster City, CA, USA) using the following protocol: 95°C for 3 min; 35 cycles of denaturation at 95°C for 30 s, annealing at 55°C for 30 s, and extension at 72°C for 45 s; and a final extension at 72°C for 7 min. The amplified products were purified with a cycle-pure kit (Axygen, Wujiang, China) and sequenced using an ABI 3500 DNA sequencer (Applied Biosystems). DNA sequences were analyzed with a genomic reference sequence on NCBI BLAST. The mutation was named according to the recommendations of the Human Genomic Variation Society (HGVS: http://www.hgvs.org/).

**Table 2:** Primer sequences used in this work

Primer name	Sequence	PCR product
TYR CD1 AF	GCT GGA GGT GGG AGT GGT ATT	459bp
TYR CD1 AR	GTC CCC AAA AGC CAA ACT TG	
TYR CD1 BF	AAT GCA CCA CTT GGG CCT C	536bp
TYR CD1 BR	TCC CGC CAG TCC CAA TAT G	
TYR CD1 CF	CAA CAC CCA TGT TTA ACG ACA	475bp
TYR CD1 CR	CAT TGA GAG TTC TTA ACA GGG C	
TYR CD2 F	GAT TTC TCA GAA CAT ATC CCT G	526bp
TYR CD2 R	AGC TAG GGT CAT TGT CGA TAT	
TYR CD3 F	AGA GTC TCA ATA CGG AAT GAA TT	519bp
TYR CD3 R	GTA TCC TGC CTA ATC CAC CTT	
TYR CD4 F	CTG TTT CCA ATT TAG TTT TAT AC	790bp
TYR CD4 R	TAC AAA ATG GCC TAT GTT AAG C	
TYR CD5 F	TGT CTA CTC CAA AGG ACT GT	924bp
TYR CD5 R	GGC ACT TAG CTG GAT GTG TT	
TYR CD4 Sequencing F	CTC CAG ATT TTA ATA TAT GCC	348bp
TYR CD4 Sequencing R	GTG TTA TCT CAA AAT AAA TTG G	
TYR CD5 Sequencing F	GAT GGT GAT CGT AAC AAT GG	311bp
TYR CD5 Sequencing R	TTT GGC CCT ACT CTA TTG CC	
OCA2 CD1 F	CGA AGA AGC AAC CTT CCT ATT GTA C	490bp
OCA2 CD1 R	CTA AGC CAG GAA AGT GAT CTA ATG C	
OCA2 CD2 F	ATT CTT GAA TCT AGC ACC TGA GTG C	306bp
OCA2 CD2 R	TGT CAA GGA TCT GGC AGA GGT TA	
OCA2 CD3 F	ACC CAT TCC CAC CAG TAT GAG AGT	456bp
OCA2 CD3 R	CAA AAC TCA TCC TCT TCT TCA CGC	
OCA2 CD4 F	TGA GAT GGA AGT TAC TCA AGG CTG	285bp
OCA2 CD4 R	AGA CAG TCA GAG AAT CAG GCG AAG	
OCA2 CD5 F	AGT AGC CCC ATC ATC ACA TCT GTT	298bp
OCA2 CD5 R	AAA TTC GAG TGG TAA TGG CCT GT	
OCA2 CD6 F	TTC TTC ACA CAC TGT CAG AGG AGG	382bp
OCA2 CD6 R	GAA TTG ACT AAG AAT GGT GTC CTC G	
OCA2 CD7 F	AAC AAA TAC CTA GAC CGA GCA GTG	242bp
OCA2 CD7 R	TAT AGG TCA GAC TCC TTT AAA CGC A	
OCA2 CD8 F	GCT GTG AGA TTG GGC GTT GG	461bp
OCA2 CD8 R	GCA AAT ATT CCT GTA TGG TTC CCT T	
OCA2 CD9 F	GCC TGA AAC ATC AAG ACC CAT	460bp
OCA2 CD9 R	CCT TTC CTC CAC CAC GAT G	
OCA2 CD10 F	CAG CGA TAT AAT CCA ACT TCA AAG G	355bp
OCA2 CD10 R	GCA CTA ACA CTT CTC AGT CAA GCC	
OCA2 CD11 F	TGT AAG GGA TCA TGC TGA TGT CG	387bp
OCA2 CD11 R	CAC AAC GAT TCA ACC TGA GTA CCC	
OCA2 CD12 F	AAT GTT AGT TTG GCT CCC TGT TCT T	330bp
OCA2 CD12 R	TCA TGC ACC TGA GAA TGG AAC C	
OCA2 CD13 F	ACT CTG GAA AGG AAT GTA ACT CTC G	491bp
OCA2 CD13 R	CTT GAG ATG CCC AGT AGC ACT TAC	
OCA2 CD14 F	ATC CAC CCA CCT CGG AAA GT	329bp
OCA2 CD14 R	AGC ATC CAG CAA CCC ATC AA	
OCA2 CD15 F	GTC TCG AGT GTG TGT CTG CTC TGT C	425bp
OCA2 CD15 R	TGC AGA GCT CAG TGA GGG TTA GAT A	
OCA2 CD16 F	ACA CTC CTT TCA TCA TTC AGG TCA T	423bp
OCA2 CD16 R	AAC CTC AAC GTC TTG TGT ATA ACC A	
OCA2 CD17 F	CTG TCG TGA TTC CAG TTG CGT AG	489bp
OCA2 CD17 R	CAG TGC CCA CTC TAT ATT CCT CCT C	
OCA2 CD18 F	GAG GTA CAA GAA CAT AGG CAT GAA T	552bp
OCA2 CD18 R	AAA TCT CTC AGT GGC TAA GGT AAA G	
OCA2 CD19 F	TCT GGG CCT ACC TTA TGT TCA CG	324bp
OCA2 CD19 R	CAT CTC TGG GCT GCA CAG GAT AG	
OCA2 CD20 F	CTA TGT CTG CCT TGG TCT CGT GAT	379bp
OCA2 CD20 R	CTC TGC TCA CTT TCG TCC TCT ACA C	
OCA2 CD21 F	GGT TTC TTT CCA CAA ATC TTA TGC T	341bp
OCA2 CD21 R	CAT CCA GAC TCT CCT TCA TTT GCT	
OCA2 CD22 F	CAA ATC AAA GCC TGT GAG ATG ATC T	326bp
OCA2 CD22 R	CTC CCC TAC ACC ACA GTC TCT CTA C	
OCA2 CD23 F	GAT GAA CAA ACA GAG GCT CCA	477bp
OCA2 CD23 R	TAG CAT CTC CAG GGT AAG CAC	
SLC45A2 CD1 F	CTG ACC ATC TCT GTT GGT TGC TC	594bp
SLC45A2 CD1 R	CTA GGA AAG GTC AAA CAC ATG AAC A	
SLC45A2 CD2 F	GGA AGA TGA TTT TAT GGC AAG AAG T	357bp
SLC45A2 CD2 R	CGT GTA GAG ACA CTG GAT GGC TT	
SLC45A2 CD3 F	CCC ACT GAA GGG GAG TGT CTA TG	518bp
SLC45A2 CD3 R	CCA TGA AAC TCT TCT CGT CAA ACA G	
SLC45A2 CD4 F	ACA CTT TGT GTG ATG GCT GAC TGA C	358bp
SLC45A2 CD4 R	ACT GTG CCA ATC TTA GAG GAT AGC C	
SLC45A2 CD5 F	GAC ATT TGC TCC CCA GAG GT	451bp
SLC45A2 CD5 R	ACC CAC TGA TTC CAA GAG CAA A	
SLC45A2 CD6 F	CCA CAG ATA AGG GGA TTC TTT TGT T	449bp
SLC45A2 CD6 R	TTC CAG CTC TGC TCT ACA CAT TGC	
SLC45A2 CD7 F	ATC CAC GAA GCC AAA GGT A	459bp
SLC45A2 CD7 R	GAA ATC ACA ATA GTG GGC GT	
